# Supplementary figures and images for: ELOVL2-AS1 inhibits migration of triple negative breast cancer
Source: PeerJ. 2022 Apr 14;10:e13264. doi: 10.7717/peerj.13264 (PMC9013481; doi:10.7717/peerj.13264)

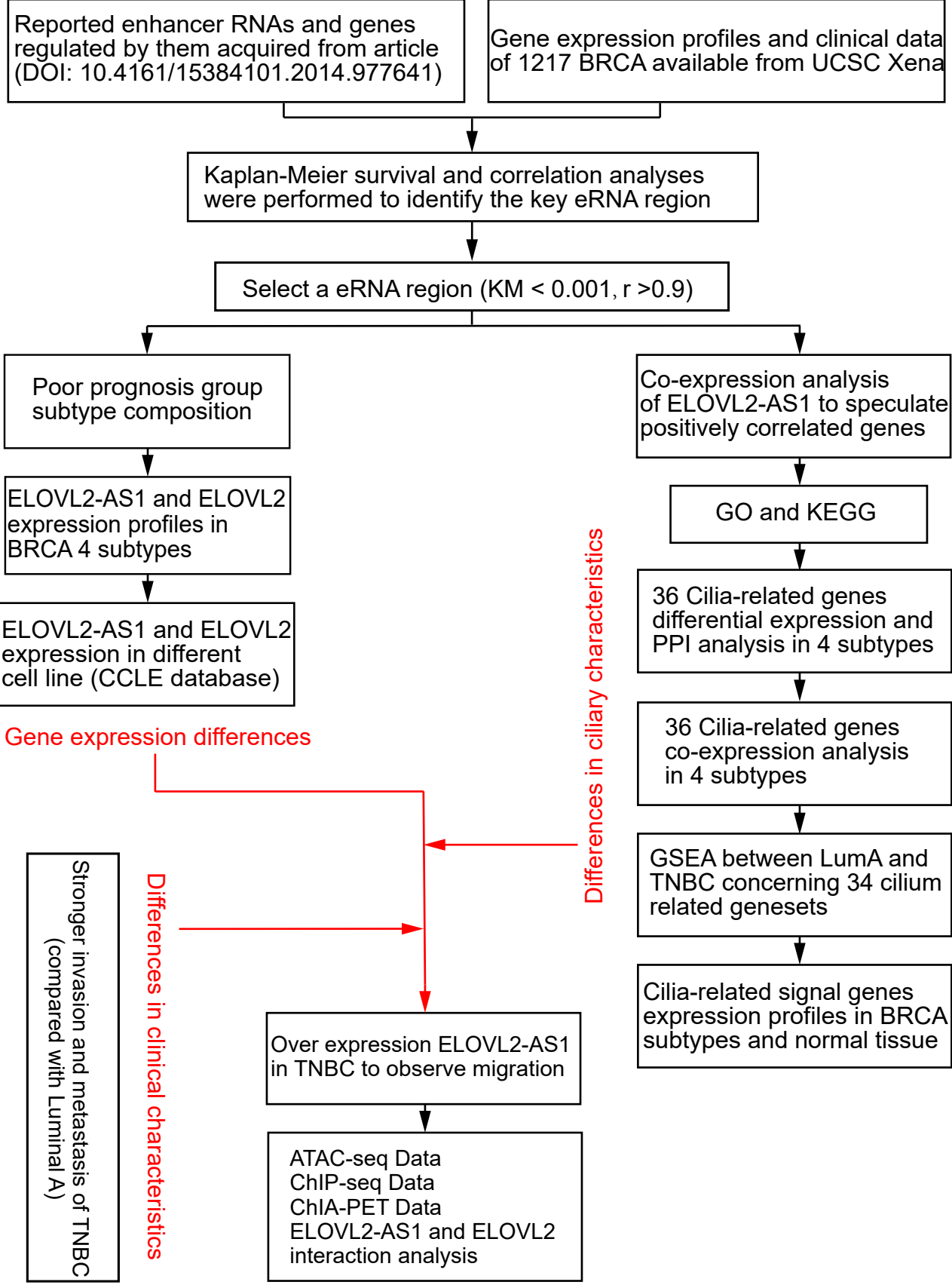

Supplement: Supplemental Information 1 [file peerj-10-13264-s001.pdf]

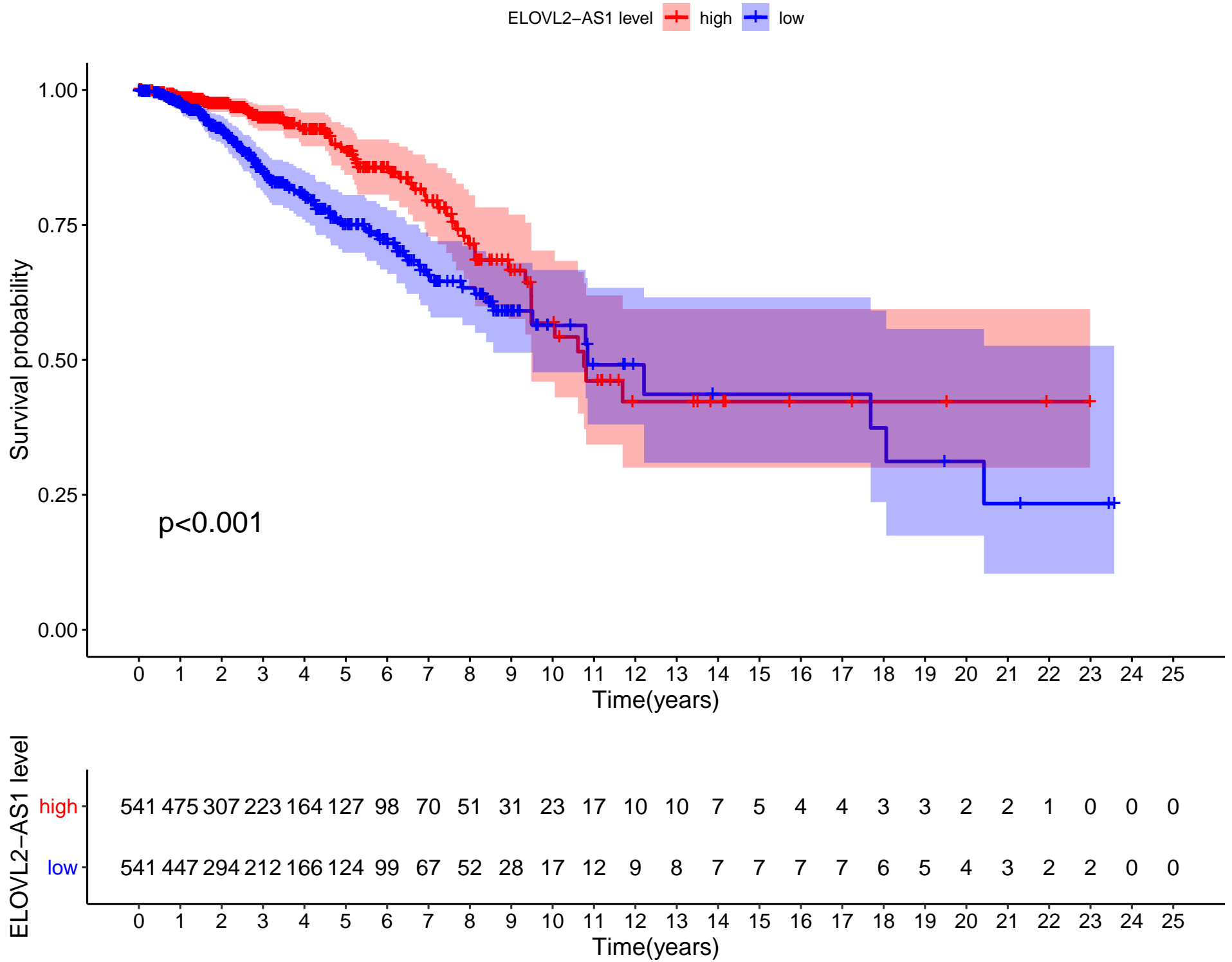

Supplement: Supplemental Information 2 [file peerj-10-13264-s002.pdf]

A

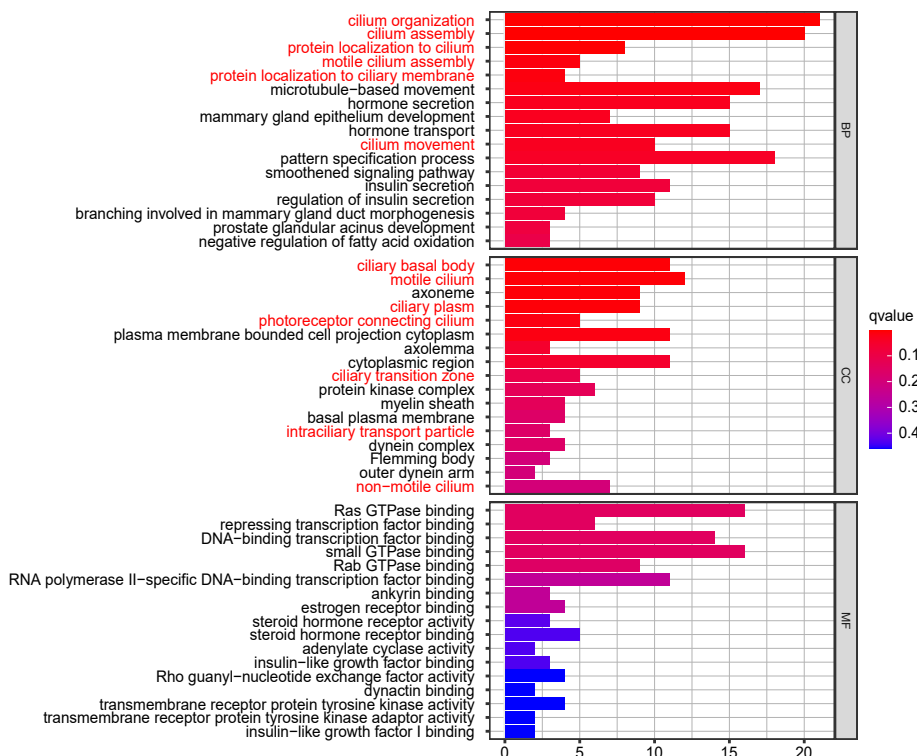

B

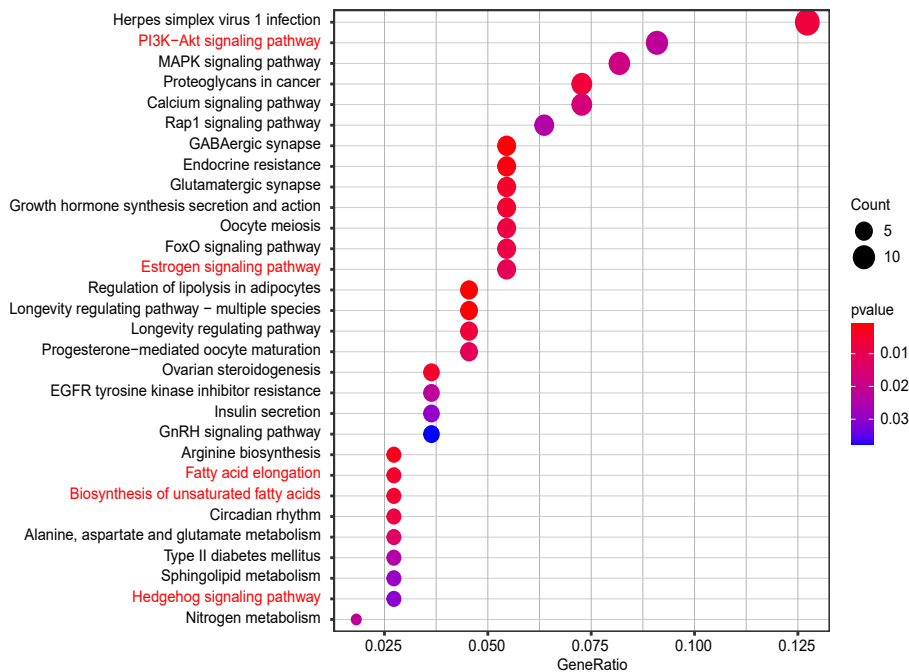

Supplement: Supplemental Information 3 [file peerj-10-13264-s003.pdf]
