# Supplementary material for: ELOVL2-AS1 inhibits migration of triple negative breast cancer
Source: PeerJ. 2022 Apr 14;10:e13264. doi: 10.7717/peerj.13264 (PMC9013481; doi:10.7717/peerj.13264)
Supplement: Supplemental Information 4 [file peerj-10-13264-s004.pdf]

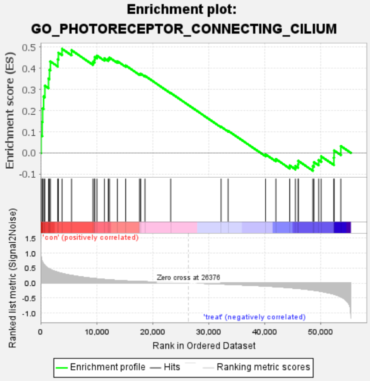
Enrichment plot:
GO_PHOTORECEPTOR_CONNECTING_CILIUM
Enrichment score (ES)
'con' (positively correlated)
Zero cross at 26376
'treat' (negatively correlated)
Ranked list metric (Signal2Noise)
Rank in Ordered Dataset
Enrichment profile
Hits
Ranking metric scores

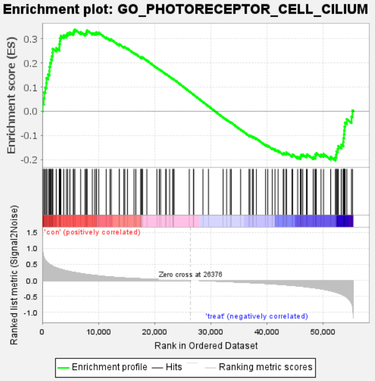
Enrichment plot: GO_PHOTORECEPTOR_CELL_CILIUM
Enrichment score (ES)
'con' (positively correlated)
Zero cross at 26376
'treat' (negatively correlated)
Ranked list metric (Signal2Noise)
Rank in Ordered Dataset
Enrichment profile
Hits
Ranking metric scores

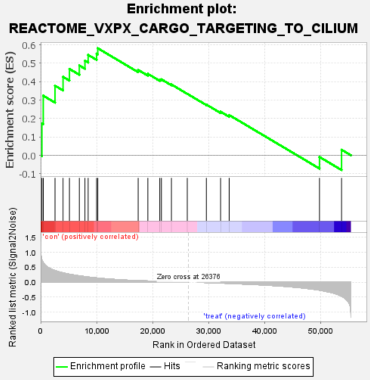
Enrichment plot:
REACTOME_VXPX_CARGO_TARGETING_TO_CILIUM
Enrichment score (ES)
'con' (positively correlated)
Zero cross at 26376
'treat' (negatively correlated)
Ranked list metric (Signal2Noise)
Rank in Ordered Dataset
Enrichment profile
Hits
Ranking metric scores

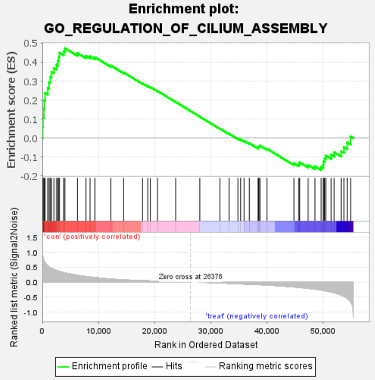
Enrichment plot:
GO_REGULATION_OF_CILIUM_ASSEMBLY
Enrichment score (ES)
'con' (positively correlated)
Zero cross at 26376
'treat' (negatively correlated)
Ranked list metric (Signal2Noise)
Rank in Ordered Dataset
Enrichment profile
Hits
Ranking metric scores

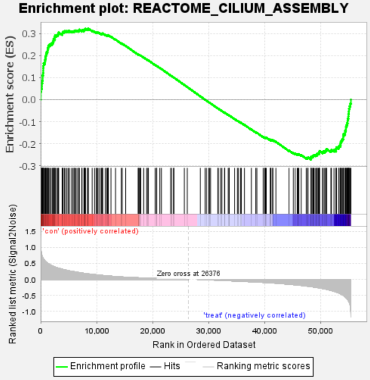
Enrichment plot: REACTOME_CILIUM_ASSEMBLY
Enrichment score (ES)
'con' (positively correlated)
Zero cross at 26376
'treat' (negatively correlated)
Ranked list metric (Signal2Noise)
Rank in Ordered Dataset
Enrichment profile
Hits
Ranking metric scores

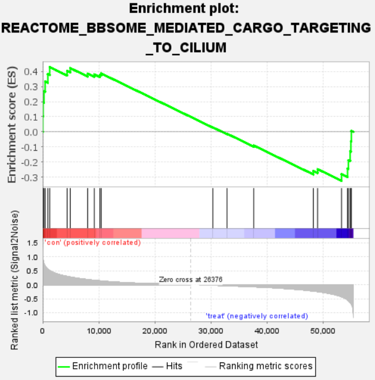
Enrichment plot:
REACTOME_BBSOME_MEDIATED_CARGO_TARGETING
_TO_CILIUM
Enrichment score (ES)
'con' (positively correlated)
Zero cross at 26376
'treat' (negatively correlated)
Ranked list metric (Signal2Noise)
Rank in Ordered Dataset
Enrichment profile
Hits
Ranking metric scores

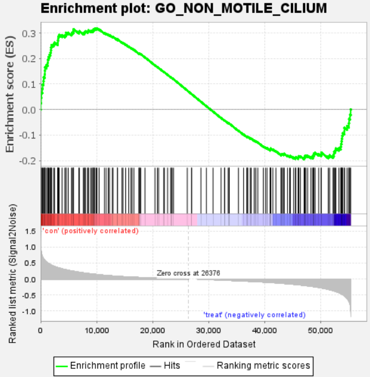
Enrichment plot: GO_NON_MOTILE_CILIUM
Enrichment score (ES)
'con' (positively correlated)
Zero cross at 26376
'treat' (negatively correlated)
Ranked list metric (Signal2Noise)
Rank in Ordered Dataset
Enrichment profile
Hits
Ranking metric scores

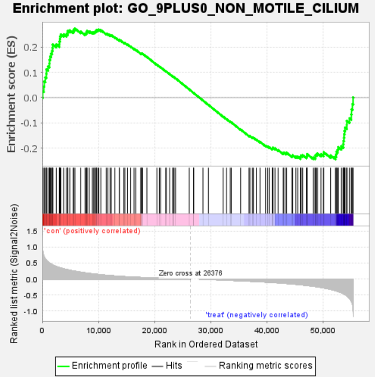
Enrichment plot: GO_9PLUS0_NON_MOTILE_CILIUM
Enrichment score (ES)
'con' (positively correlated)
Zero cross at 26376
'treat' (negatively correlated)
Ranked list metric (Signal2Noise)
Rank in Ordered Dataset
Enrichment profile
Hits
Ranking metric scores
