# Supplementary material for: ELOVL2-AS1 inhibits migration of triple negative breast cancer
Source: PeerJ. 2022 Apr 14;10:e13264. doi: 10.7717/peerj.13264 (PMC9013481; doi:10.7717/peerj.13264)
Supplement: Supplemental Information 5 [file peerj-10-13264-s005.docx]

**Table S1:**

| eRNA region | KM | Target | cor | corPval |
| --- | --- | --- | --- | --- |
| AP003059.2 | 0.000545 | ME3 | 0.733024 | 1.21E-186 |
| AP003059.2 | 0.000545 | PRSS23 | 0.476012 | 1.65E-63 |
| ELOVL2-AS1 | 0.000759 | ELOVL2 | 0.925021 | 0 |
| LINC00987 | 0.002302 | A2M | 0.662404 | 2.15E-140 |
| LINC00987 | 0.002302 | PZP | 0.583514 | 1.01E-101 |
| MAL2 | 0.002315 | MAL2 | 1 | 0 |
| GNG12-AS1 | 0.002769 | DIRAS3 | 0.501058 | 3.17E-71 |
| GNG12-AS1 | 0.002769 | GNG12 | 0.719041 | 2.34E-176 |
| LINC01087 | 0.004236 | CCDC74A | 0.602353 | 5.56E-110 |
| LINC01087 | 0.004236 | POTEKP | 0.973322 | 0 |
| LINC02677 | 0.00576 | CALML3 | 0.543985 | 4.83E-86 |
| STK3 | 0.014896 | NIPAL2 | 0.408393 | 0 |
| AC025164.2 | 0.015314 | BTG1 | 0.544037 | 4.62E-86 |
| LINC00689 | 0.01634 | VIPR2 | 0.568718 | 1.33E-95 |
| AL139246.2 | 0.017244 | TNFRSF14 | 0.483889 | 7.22E-66 |
| AC116351.1 | 0.017942 | NKD2 | 0.435967 | 1.97E-52 |
| IFNG-AS1 | 0.019075 | IFNG | 0.616189 | 2.08E-116 |
| AC008663.1 | 0.020307 | BOD1 | 0.428087 | 2.02E-50 |
| IQANK1 | 0.023081 | FAM83H | 0.715587 | 0 |
| LINC00472 | 0.023142 | OGFRL1 | 0.469269 | 1.54E-61 |
| AL034417.3 | 0.026302 | ERRFI1 | 0.52035 | 1.27E-77 |
| LINC01863 | 0.029878 | BOD1 | 0.40188 | 4.19E-44 |
| AC008957.1 | 0.030033 | SLC1A3 | 0.501856 | 1.75E-71 |
| TP53TG1 | 0.030116 | CROT | 0.649579 | 0 |
| AC008663.3 | 0.030904 | BOD1 | 0.44197 | 5.33E-54 |
| LNCAROD | 0.031642 | DKK1 | 0.494083 | 5.18E-69 |
| LRRC8C-DT | 0.035066 | LRRC8C | 0.775577 | 2.07E-222 |
| LINC01122 | 0.036059 | FANCL | 0.479311 | 1.72E-64 |
| AC105942.1 | 0.03809 | CNN3 | 0.758388 | 0 |
| SLC25A24P1 | 0.039791 | NBPF4 | 0.882274 | 0 |
| SLC25A24P1 | 0.039791 | NBPF6 | 0.83113 | 3.99E-283 |
| WAKMAR2 | 0.042401 | TNFAIP3 | 0.790534 | 0 |
| LINC02523 | 0.044652 | HEY2 | 0.41297 | 1.04E-46 |
| AC004083.1 | 0.048782 | TMEM64 | 0.555081 | 3.12E-90 |
| LINC01010 | 0.049001 | SGK1 | 0.500092 | 6.46E-71 |

35 target pairs
