# Supplementary material for: ELOVL2-AS1 inhibits migration of triple negative breast cancer
Source: PeerJ. 2022 Apr 14;10:e13264. doi: 10.7717/peerj.13264 (PMC9013481; doi:10.7717/peerj.13264)
Supplement: Supplemental Information 6 [file peerj-10-13264-s006.docx]

**Table S2：**

429 genes are positively correlated with the expression of ELOVL2-AS1

| eRNA region | gene | cor | pvalue |
| --- | --- | --- | --- |
| ELOVL2-AS1 | ELOVL2 | 0.925 | 0 |
| ELOVL2-AS1 | AL121955.1 | 0.652 | 1.40E-134 |
| ELOVL2-AS1 | LINC01016 | 0.634 | 5.92E-125 |
| ELOVL2-AS1 | NXNL2 | 0.628 | 2.48E-122 |
| ELOVL2-AS1 | GREB1 | 0.594 | 2.37E-106 |
| ELOVL2-AS1 | THSD4 | 0.578 | 1.92E-99 |
| ELOVL2-AS1 | MAPT | 0.577 | 3.52E-99 |
| ELOVL2-AS1 | MYB | 0.575 | 2.82E-98 |
| ELOVL2-AS1 | PREX1 | 0.565 | 3.69E-94 |
| ELOVL2-AS1 | UGCG | 0.563 | 2.37E-93 |
| ELOVL2-AS1 | SYTL4 | 0.556 | 9.42E-91 |
| ELOVL2-AS1 | KDM4B | 0.554 | 7.52E-90 |
| ELOVL2-AS1 | TMEM26 | 0.553 | 1.85E-89 |
| ELOVL2-AS1 | AL031429.2 | 0.55 | 3.46E-88 |
| ELOVL2-AS1 | RAB6C | 0.547 | 3.34E-87 |
| ELOVL2-AS1 | PTGER3 | 0.546 | 1.27E-86 |
| ELOVL2-AS1 | AC008663.1 | 0.546 | 1.13E-86 |
| ELOVL2-AS1 | LINC02130 | 0.546 | 7.01E-87 |
| ELOVL2-AS1 | WDR19 | 0.542 | 1.97E-85 |
| ELOVL2-AS1 | MAPT-IT1 | 0.542 | 2.46E-85 |
| ELOVL2-AS1 | ADCY1 | 0.54 | 1.95E-84 |
| ELOVL2-AS1 | MAPT-AS1 | 0.54 | 1.12E-84 |
| ELOVL2-AS1 | CT62 | 0.535 | 1.10E-82 |
| ELOVL2-AS1 | AC011498.4 | 0.534 | 2.38E-82 |
| ELOVL2-AS1 | INPP4B | 0.531 | 2.99E-81 |
| ELOVL2-AS1 | CA12 | 0.529 | 1.11E-80 |
| ELOVL2-AS1 | THSD4-AS1 | 0.529 | 1.68E-80 |
| ELOVL2-AS1 | IL6ST | 0.529 | 1.84E-80 |
| ELOVL2-AS1 | CLSTN2 | 0.528 | 2.60E-80 |
| ELOVL2-AS1 | IGF1R | 0.527 | 8.69E-80 |
| ELOVL2-AS1 | STC2 | 0.527 | 5.40E-80 |
| ELOVL2-AS1 | AC103760.1 | 0.525 | 3.15E-79 |
| ELOVL2-AS1 | C5AR2 | 0.525 | 4.07E-79 |
| ELOVL2-AS1 | AFF3 | 0.523 | 1.19E-78 |
| ELOVL2-AS1 | RAB6C-AS1 | 0.519 | 3.76E-77 |
| ELOVL2-AS1 | PGR | 0.518 | 5.93E-77 |
| ELOVL2-AS1 | SCUBE2 | 0.517 | 1.92E-76 |
| ELOVL2-AS1 | TMEM26-AS1 | 0.517 | 1.75E-76 |
| ELOVL2-AS1 | Z97633.1 | 0.517 | 1.33E-76 |
| ELOVL2-AS1 | GFRA1 | 0.516 | 2.94E-76 |
| ELOVL2-AS1 | PLIN5 | 0.516 | 3.78E-76 |
| ELOVL2-AS1 | SUSD3 | 0.515 | 7.40E-76 |
| ELOVL2-AS1 | PDZK1 | 0.515 | 1.05E-75 |
| ELOVL2-AS1 | WNK4 | 0.514 | 2.31E-75 |
| ELOVL2-AS1 | TBC1D9 | 0.514 | 1.38E-75 |
| ELOVL2-AS1 | CELSR1 | 0.514 | 1.38E-75 |
| ELOVL2-AS1 | PARD6B | 0.514 | 1.26E-75 |
| ELOVL2-AS1 | RBM24 | 0.513 | 4.67E-75 |
| ELOVL2-AS1 | AC008663.3 | 0.513 | 4.08E-75 |
| ELOVL2-AS1 | ZNF442 | 0.512 | 8.99E-75 |
| ELOVL2-AS1 | AC124067.4 | 0.511 | 1.79E-74 |
| ELOVL2-AS1 | BCL2 | 0.51 | 4.28E-74 |
| ELOVL2-AS1 | CST9 | 0.508 | 1.82E-73 |
| ELOVL2-AS1 | KIF12 | 0.507 | 3.62E-73 |
| ELOVL2-AS1 | RAD17P1 | 0.507 | 3.15E-73 |
| ELOVL2-AS1 | ARSG | 0.504 | 3.16E-72 |
| ELOVL2-AS1 | TTC39A-AS1 | 0.504 | 3.77E-72 |
| ELOVL2-AS1 | ESR1 | 0.504 | 4.91E-72 |
| ELOVL2-AS1 | LINC02747 | 0.503 | 6.85E-72 |
| ELOVL2-AS1 | NR2E3 | 0.503 | 7.23E-72 |
| ELOVL2-AS1 | ANKRA2 | 0.503 | 9.91E-72 |
| ELOVL2-AS1 | IFT140 | 0.501 | 2.68E-71 |
| ELOVL2-AS1 | AC092667.1 | 0.5 | 7.40E-71 |
| ELOVL2-AS1 | IGDCC3 | 0.5 | 7.29E-71 |
| ELOVL2-AS1 | RALGPS2 | 0.499 | 1.25E-70 |
| ELOVL2-AS1 | TTC39A | 0.499 | 1.88E-70 |
| ELOVL2-AS1 | FRS2 | 0.499 | 1.82E-70 |
| ELOVL2-AS1 | ABAT | 0.499 | 1.55E-70 |
| ELOVL2-AS1 | SLC39A6 | 0.496 | 9.84E-70 |
| ELOVL2-AS1 | CELSR2 | 0.496 | 1.34E-69 |
| ELOVL2-AS1 | RERG-IT1 | 0.494 | 4.32E-69 |
| ELOVL2-AS1 | TPBG | 0.493 | 8.66E-69 |
| ELOVL2-AS1 | ZNF703 | 0.493 | 1.44E-68 |
| ELOVL2-AS1 | TNRC18P1 | 0.493 | 9.62E-69 |
| ELOVL2-AS1 | AC096733.3 | 0.491 | 5.58E-68 |
| ELOVL2-AS1 | AC020917.4 | 0.491 | 4.28E-68 |
| ELOVL2-AS1 | WWP1 | 0.491 | 4.95E-68 |
| ELOVL2-AS1 | ZNF44 | 0.49 | 1.14E-67 |
| ELOVL2-AS1 | GRPR | 0.49 | 1.14E-67 |
| ELOVL2-AS1 | MYB-AS1 | 0.49 | 1.23E-67 |
| ELOVL2-AS1 | SFXN2 | 0.489 | 1.72E-67 |
| ELOVL2-AS1 | ZNF552 | 0.489 | 1.90E-67 |
| ELOVL2-AS1 | LINC02568 | 0.488 | 3.45E-67 |
| ELOVL2-AS1 | TPSG1 | 0.487 | 6.16E-67 |
| ELOVL2-AS1 | BHLHE40 | 0.485 | 2.52E-66 |
| ELOVL2-AS1 | CCDC170 | 0.484 | 7.35E-66 |
| ELOVL2-AS1 | MRTFB | 0.482 | 2.58E-65 |
| ELOVL2-AS1 | LINC01087 | 0.481 | 5.44E-65 |
| ELOVL2-AS1 | POTEKP | 0.481 | 5.80E-65 |
| ELOVL2-AS1 | AC078993.1 | 0.48 | 1.38E-64 |
| ELOVL2-AS1 | PLPPR3 | 0.48 | 1.16E-64 |
| ELOVL2-AS1 | RABEP1 | 0.479 | 2.72E-64 |
| ELOVL2-AS1 | RERG | 0.479 | 1.86E-64 |
| ELOVL2-AS1 | AC093866.1 | 0.479 | 2.31E-64 |
| ELOVL2-AS1 | AZU1 | 0.477 | 8.24E-64 |
| ELOVL2-AS1 | SLC22A5 | 0.476 | 1.23E-63 |
| ELOVL2-AS1 | ELP2 | 0.476 | 1.48E-63 |
| ELOVL2-AS1 | ACADSB | 0.476 | 1.35E-63 |
| ELOVL2-AS1 | RUNDC1 | 0.475 | 2.94E-63 |
| ELOVL2-AS1 | CCDC74B | 0.475 | 3.02E-63 |
| ELOVL2-AS1 | LINC00504 | 0.475 | 2.72E-63 |
| ELOVL2-AS1 | NKAIN1 | 0.475 | 2.35E-63 |
| ELOVL2-AS1 | ZNF92 | 0.475 | 3.01E-63 |
| ELOVL2-AS1 | CCDC30 | 0.475 | 2.83E-63 |
| ELOVL2-AS1 | GRAMD4P8 | 0.474 | 7.20E-63 |
| ELOVL2-AS1 | PLAC9P1 | 0.473 | 1.18E-62 |
| ELOVL2-AS1 | CMYA5 | 0.473 | 1.72E-62 |
| ELOVL2-AS1 | TPTE2P2 | 0.472 | 2.44E-62 |
| ELOVL2-AS1 | SRARP | 0.472 | 2.03E-62 |
| ELOVL2-AS1 | SIAH2-AS1 | 0.472 | 3.39E-62 |
| ELOVL2-AS1 | CFAP99 | 0.471 | 4.95E-62 |
| ELOVL2-AS1 | AC008763.1 | 0.47 | 7.68E-62 |
| ELOVL2-AS1 | MAG | 0.47 | 1.20E-61 |
| ELOVL2-AS1 | HHAT | 0.47 | 1.05E-61 |
| ELOVL2-AS1 | SLC7A13 | 0.47 | 8.21E-62 |
| ELOVL2-AS1 | AC007780.1 | 0.47 | 9.11E-62 |
| ELOVL2-AS1 | ZNF844 | 0.47 | 1.02E-61 |
| ELOVL2-AS1 | SPEF2 | 0.47 | 9.31E-62 |
| ELOVL2-AS1 | DNALI1 | 0.469 | 1.87E-61 |
| ELOVL2-AS1 | FRMPD2 | 0.468 | 4.29E-61 |
| ELOVL2-AS1 | FAM161B | 0.468 | 4.85E-61 |
| ELOVL2-AS1 | SEPSECS | 0.467 | 9.36E-61 |
| ELOVL2-AS1 | MIR3936HG | 0.467 | 5.76E-61 |
| ELOVL2-AS1 | AL356311.1 | 0.466 | 1.23E-60 |
| ELOVL2-AS1 | EVL | 0.465 | 2.74E-60 |
| ELOVL2-AS1 | ZNF763 | 0.464 | 4.50E-60 |
| ELOVL2-AS1 | RAI2 | 0.464 | 4.10E-60 |
| ELOVL2-AS1 | ZNF441 | 0.464 | 6.49E-60 |
| ELOVL2-AS1 | TTC36 | 0.464 | 4.75E-60 |
| ELOVL2-AS1 | KRT8P41 | 0.463 | 7.28E-60 |
| ELOVL2-AS1 | FAM241A | 0.463 | 1.24E-59 |
| ELOVL2-AS1 | ERICH3 | 0.462 | 1.48E-59 |
| ELOVL2-AS1 | FLNB-AS1 | 0.462 | 2.12E-59 |
| ELOVL2-AS1 | MCCD1 | 0.462 | 1.86E-59 |
| ELOVL2-AS1 | PYY | 0.462 | 1.96E-59 |
| ELOVL2-AS1 | AC083906.3 | 0.462 | 2.29E-59 |
| ELOVL2-AS1 | TPRG1 | 0.461 | 3.61E-59 |
| ELOVL2-AS1 | DNAJC12 | 0.461 | 4.43E-59 |
| ELOVL2-AS1 | LINC01863 | 0.461 | 4.11E-59 |
| ELOVL2-AS1 | GATA3 | 0.461 | 3.47E-59 |
| ELOVL2-AS1 | ABCD3 | 0.46 | 6.21E-59 |
| ELOVL2-AS1 | TIGD6 | 0.46 | 6.39E-59 |
| ELOVL2-AS1 | FLNB | 0.459 | 1.68E-58 |
| ELOVL2-AS1 | BTRC | 0.459 | 1.39E-58 |
| ELOVL2-AS1 | NPY1R | 0.459 | 9.81E-59 |
| ELOVL2-AS1 | AL449106.1 | 0.459 | 1.65E-58 |
| ELOVL2-AS1 | AL158206.1 | 0.458 | 2.92E-58 |
| ELOVL2-AS1 | ZNF396 | 0.457 | 4.44E-58 |
| ELOVL2-AS1 | FGD3 | 0.457 | 4.49E-58 |
| ELOVL2-AS1 | SLC16A6 | 0.457 | 4.92E-58 |
| ELOVL2-AS1 | SEMA3F | 0.457 | 6.16E-58 |
| ELOVL2-AS1 | ZNF587 | 0.457 | 5.42E-58 |
| ELOVL2-AS1 | STH | 0.456 | 9.90E-58 |
| ELOVL2-AS1 | CFAP69 | 0.456 | 9.18E-58 |
| ELOVL2-AS1 | VAV3 | 0.456 | 8.09E-58 |
| ELOVL2-AS1 | CCDC74A | 0.456 | 1.18E-57 |
| ELOVL2-AS1 | GRIK4 | 0.455 | 1.94E-57 |
| ELOVL2-AS1 | AP003065.1 | 0.455 | 1.82E-57 |
| ELOVL2-AS1 | KCNK15 | 0.455 | 1.69E-57 |
| ELOVL2-AS1 | TRIM45 | 0.454 | 2.45E-57 |
| ELOVL2-AS1 | KCNH1 | 0.454 | 2.50E-57 |
| ELOVL2-AS1 | KANSL3 | 0.454 | 3.11E-57 |
| ELOVL2-AS1 | FSIP1 | 0.453 | 4.97E-57 |
| ELOVL2-AS1 | RNF213-AS1 | 0.453 | 4.33E-57 |
| ELOVL2-AS1 | CXXC5 | 0.453 | 6.65E-57 |
| ELOVL2-AS1 | NRIP1 | 0.453 | 7.62E-57 |
| ELOVL2-AS1 | ZNF799 | 0.452 | 8.66E-57 |
| ELOVL2-AS1 | AL133297.2 | 0.452 | 1.13E-56 |
| ELOVL2-AS1 | APH1B | 0.452 | 1.05E-56 |
| ELOVL2-AS1 | AC064799.2 | 0.452 | 8.10E-57 |
| ELOVL2-AS1 | DRC3 | 0.452 | 1.25E-56 |
| ELOVL2-AS1 | MS4A8 | 0.452 | 8.04E-57 |
| ELOVL2-AS1 | GLUL | 0.451 | 2.74E-56 |
| ELOVL2-AS1 | KLHDC1 | 0.451 | 1.80E-56 |
| ELOVL2-AS1 | ZMYND10 | 0.45 | 4.72E-56 |
| ELOVL2-AS1 | PCBD2 | 0.45 | 3.15E-56 |
| ELOVL2-AS1 | TLE3 | 0.45 | 3.43E-56 |
| ELOVL2-AS1 | BBS4 | 0.45 | 4.27E-56 |
| ELOVL2-AS1 | CACNA1D | 0.45 | 3.98E-56 |
| ELOVL2-AS1 | TMEM229B | 0.449 | 5.82E-56 |
| ELOVL2-AS1 | NAT1 | 0.449 | 6.16E-56 |
| ELOVL2-AS1 | SYTL5 | 0.449 | 8.66E-56 |
| ELOVL2-AS1 | TTC8 | 0.449 | 7.10E-56 |
| ELOVL2-AS1 | CST9L | 0.449 | 9.14E-56 |
| ELOVL2-AS1 | STMND1 | 0.449 | 6.72E-56 |
| ELOVL2-AS1 | DNAAF1 | 0.449 | 6.45E-56 |
| ELOVL2-AS1 | ANXA9 | 0.449 | 7.91E-56 |
| ELOVL2-AS1 | AC093297.2 | 0.448 | 1.51E-55 |
| ELOVL2-AS1 | AC012467.1 | 0.447 | 2.34E-55 |
| ELOVL2-AS1 | AC080112.4 | 0.447 | 1.87E-55 |
| ELOVL2-AS1 | FGFR3 | 0.447 | 2.14E-55 |
| ELOVL2-AS1 | LONRF2 | 0.447 | 2.19E-55 |
| ELOVL2-AS1 | SH3D21 | 0.446 | 6.10E-55 |
| ELOVL2-AS1 | KCNJ11 | 0.446 | 3.83E-55 |
| ELOVL2-AS1 | MLPH | 0.446 | 5.34E-55 |
| ELOVL2-AS1 | NUDT6 | 0.445 | 9.55E-55 |
| ELOVL2-AS1 | IRS1 | 0.445 | 1.00E-54 |
| ELOVL2-AS1 | MAGED2 | 0.445 | 6.52E-55 |
| ELOVL2-AS1 | MINDY1 | 0.444 | 1.49E-54 |
| ELOVL2-AS1 | WDR93 | 0.444 | 1.22E-54 |
| ELOVL2-AS1 | AC010326.4 | 0.444 | 1.17E-54 |
| ELOVL2-AS1 | KLHDC2 | 0.444 | 1.77E-54 |
| ELOVL2-AS1 | MRPS30-DT | 0.444 | 1.64E-54 |
| ELOVL2-AS1 | PCP2 | 0.444 | 1.26E-54 |
| ELOVL2-AS1 | AL133387.1 | 0.443 | 3.10E-54 |
| ELOVL2-AS1 | LYPD6 | 0.443 | 2.39E-54 |
| ELOVL2-AS1 | AC008663.2 | 0.443 | 2.42E-54 |
| ELOVL2-AS1 | AL121578.2 | 0.443 | 2.23E-54 |
| ELOVL2-AS1 | AC005225.4 | 0.443 | 2.91E-54 |
| ELOVL2-AS1 | ZNF823 | 0.442 | 4.43E-54 |
| ELOVL2-AS1 | TTC6 | 0.442 | 6.34E-54 |
| ELOVL2-AS1 | NOVA1 | 0.442 | 3.91E-54 |
| ELOVL2-AS1 | AC037198.2 | 0.442 | 6.76E-54 |
| ELOVL2-AS1 | POLI | 0.442 | 4.05E-54 |
| ELOVL2-AS1 | AC012313.6 | 0.441 | 1.12E-53 |
| ELOVL2-AS1 | PRKAB1 | 0.441 | 1.24E-53 |
| ELOVL2-AS1 | ARMT1 | 0.441 | 9.76E-54 |
| ELOVL2-AS1 | DCAF16 | 0.441 | 1.20E-53 |
| ELOVL2-AS1 | RAMP2-AS1 | 0.441 | 8.67E-54 |
| ELOVL2-AS1 | PPM1J | 0.441 | 7.98E-54 |
| ELOVL2-AS1 | MYOZ3 | 0.441 | 1.07E-53 |
| ELOVL2-AS1 | AC012313.2 | 0.441 | 1.12E-53 |
| ELOVL2-AS1 | UBXN10 | 0.441 | 7.90E-54 |
| ELOVL2-AS1 | POC5 | 0.44 | 1.76E-53 |
| ELOVL2-AS1 | MAK | 0.44 | 1.81E-53 |
| ELOVL2-AS1 | ADCY9 | 0.44 | 1.73E-53 |
| ELOVL2-AS1 | DCDC1 | 0.439 | 3.49E-53 |
| ELOVL2-AS1 | KIAA0232 | 0.439 | 2.62E-53 |
| ELOVL2-AS1 | ETFBKMT | 0.439 | 4.04E-53 |
| ELOVL2-AS1 | MYRIP | 0.439 | 2.73E-53 |
| ELOVL2-AS1 | ERBB3 | 0.438 | 6.03E-53 |
| ELOVL2-AS1 | CPLX1 | 0.438 | 5.66E-53 |
| ELOVL2-AS1 | BBOF1 | 0.438 | 5.85E-53 |
| ELOVL2-AS1 | SLC24A1 | 0.438 | 4.96E-53 |
| ELOVL2-AS1 | MAP3K1 | 0.437 | 1.35E-52 |
| ELOVL2-AS1 | CCDC191 | 0.437 | 9.62E-53 |
| ELOVL2-AS1 | SGK3 | 0.437 | 9.09E-53 |
| ELOVL2-AS1 | TRH | 0.436 | 1.82E-52 |
| ELOVL2-AS1 | PGLYRP2 | 0.435 | 3.44E-52 |
| ELOVL2-AS1 | LINC01843 | 0.435 | 4.35E-52 |
| ELOVL2-AS1 | APBB2 | 0.435 | 3.70E-52 |
| ELOVL2-AS1 | MIR29B2CHG | 0.434 | 5.98E-52 |
| ELOVL2-AS1 | SIAH2 | 0.434 | 5.95E-52 |
| ELOVL2-AS1 | GATAD1 | 0.434 | 4.82E-52 |
| ELOVL2-AS1 | AP001533.1 | 0.434 | 5.58E-52 |
| ELOVL2-AS1 | AC092162.3 | 0.434 | 4.80E-52 |
| ELOVL2-AS1 | AGR3 | 0.434 | 5.09E-52 |
| ELOVL2-AS1 | FAM47E | 0.434 | 4.94E-52 |
| ELOVL2-AS1 | COQ7 | 0.433 | 1.33E-51 |
| ELOVL2-AS1 | SREK1 | 0.433 | 1.52E-51 |
| ELOVL2-AS1 | AC098591.2 | 0.433 | 1.42E-51 |
| ELOVL2-AS1 | CHAD | 0.433 | 1.11E-51 |
| ELOVL2-AS1 | AC073573.1 | 0.432 | 2.60E-51 |
| ELOVL2-AS1 | JADE2 | 0.432 | 1.58E-51 |
| ELOVL2-AS1 | ADAMTS15 | 0.432 | 1.84E-51 |
| ELOVL2-AS1 | NXPH3 | 0.432 | 2.64E-51 |
| ELOVL2-AS1 | BRD8 | 0.432 | 1.61E-51 |
| ELOVL2-AS1 | C1orf226 | 0.431 | 3.03E-51 |
| ELOVL2-AS1 | TTLL9 | 0.43 | 5.64E-51 |
| ELOVL2-AS1 | AL133297.1 | 0.43 | 7.62E-51 |
| ELOVL2-AS1 | HECTD2 | 0.43 | 5.56E-51 |
| ELOVL2-AS1 | AGBL2 | 0.43 | 6.59E-51 |
| ELOVL2-AS1 | ZNF776 | 0.43 | 5.52E-51 |
| ELOVL2-AS1 | TRAK1 | 0.43 | 5.44E-51 |
| ELOVL2-AS1 | AC106738.2 | 0.43 | 6.53E-51 |
| ELOVL2-AS1 | TCEAL1 | 0.43 | 5.62E-51 |
| ELOVL2-AS1 | KCNH1-IT1 | 0.43 | 7.95E-51 |
| ELOVL2-AS1 | TESMIN | 0.429 | 1.45E-50 |
| ELOVL2-AS1 | GPR139 | 0.429 | 1.51E-50 |
| ELOVL2-AS1 | TADA2B | 0.429 | 9.31E-51 |
| ELOVL2-AS1 | MDM1 | 0.428 | 1.97E-50 |
| ELOVL2-AS1 | HEXIM1 | 0.428 | 2.22E-50 |
| ELOVL2-AS1 | ZNF497 | 0.428 | 2.67E-50 |
| ELOVL2-AS1 | ARL3 | 0.427 | 4.39E-50 |
| ELOVL2-AS1 | APPL2 | 0.427 | 4.04E-50 |
| ELOVL2-AS1 | LINC01856 | 0.426 | 5.44E-50 |
| ELOVL2-AS1 | RLN2 | 0.426 | 8.99E-50 |
| ELOVL2-AS1 | AMZ1 | 0.426 | 5.64E-50 |
| ELOVL2-AS1 | MIR3936 | 0.426 | 6.43E-50 |
| ELOVL2-AS1 | TNRC6C | 0.426 | 6.47E-50 |
| ELOVL2-AS1 | TTC34 | 0.426 | 7.16E-50 |
| ELOVL2-AS1 | TMEM9B-AS1 | 0.425 | 1.51E-49 |
| ELOVL2-AS1 | EXOC6 | 0.425 | 1.45E-49 |
| ELOVL2-AS1 | SLC35E2B | 0.425 | 1.35E-49 |
| ELOVL2-AS1 | PLEKHD1 | 0.425 | 9.91E-50 |
| ELOVL2-AS1 | AC018816.2 | 0.425 | 1.36E-49 |
| ELOVL2-AS1 | ZNF814 | 0.425 | 1.41E-49 |
| ELOVL2-AS1 | EFHC1 | 0.424 | 2.17E-49 |
| ELOVL2-AS1 | SLC19A2 | 0.424 | 1.73E-49 |
| ELOVL2-AS1 | LMX1B | 0.424 | 2.19E-49 |
| ELOVL2-AS1 | CSAD | 0.424 | 2.08E-49 |
| ELOVL2-AS1 | CASC1 | 0.424 | 2.65E-49 |
| ELOVL2-AS1 | MRFAP1L1 | 0.423 | 4.25E-49 |
| ELOVL2-AS1 | USP30 | 0.423 | 2.90E-49 |
| ELOVL2-AS1 | INTU | 0.423 | 3.19E-49 |
| ELOVL2-AS1 | RGS22 | 0.423 | 3.94E-49 |
| ELOVL2-AS1 | CCDC148 | 0.423 | 2.90E-49 |
| ELOVL2-AS1 | AL138889.2 | 0.423 | 4.05E-49 |
| ELOVL2-AS1 | DEGS2 | 0.422 | 7.29E-49 |
| ELOVL2-AS1 | SLC26A1 | 0.422 | 5.54E-49 |
| ELOVL2-AS1 | KCTD6 | 0.422 | 7.20E-49 |
| ELOVL2-AS1 | CSNK1G3 | 0.422 | 7.14E-49 |
| ELOVL2-AS1 | DOCK1 | 0.422 | 7.77E-49 |
| ELOVL2-AS1 | TENT2 | 0.422 | 5.53E-49 |
| ELOVL2-AS1 | NOS1AP | 0.421 | 1.52E-48 |
| ELOVL2-AS1 | AC073114.1 | 0.421 | 1.33E-48 |
| ELOVL2-AS1 | IGFALS | 0.421 | 1.39E-48 |
| ELOVL2-AS1 | STK36 | 0.421 | 1.04E-48 |
| ELOVL2-AS1 | EFCAB11 | 0.421 | 9.55E-49 |
| ELOVL2-AS1 | CCDC173 | 0.421 | 9.08E-49 |
| ELOVL2-AS1 | CR936218.2 | 0.421 | 1.09E-48 |
| ELOVL2-AS1 | AC109361.2 | 0.42 | 1.78E-48 |
| ELOVL2-AS1 | NEK10 | 0.42 | 1.84E-48 |
| ELOVL2-AS1 | PEX11A | 0.42 | 2.41E-48 |
| ELOVL2-AS1 | CACNA2D2 | 0.42 | 1.93E-48 |
| ELOVL2-AS1 | PGGT1B | 0.42 | 1.99E-48 |
| ELOVL2-AS1 | TESK2 | 0.42 | 1.99E-48 |
| ELOVL2-AS1 | PAXIP1-AS2 | 0.42 | 2.19E-48 |
| ELOVL2-AS1 | CFAP57 | 0.42 | 2.64E-48 |
| ELOVL2-AS1 | AC093297.1 | 0.42 | 2.73E-48 |
| ELOVL2-AS1 | OSCP1 | 0.42 | 1.68E-48 |
| ELOVL2-AS1 | AC093382.1 | 0.419 | 3.87E-48 |
| ELOVL2-AS1 | GATA3-AS1 | 0.419 | 2.77E-48 |
| ELOVL2-AS1 | SERPINA5 | 0.418 | 6.76E-48 |
| ELOVL2-AS1 | DNAL1 | 0.417 | 8.66E-48 |
| ELOVL2-AS1 | SUOX | 0.417 | 1.08E-47 |
| ELOVL2-AS1 | PCSK4 | 0.417 | 1.29E-47 |
| ELOVL2-AS1 | RNU6-813P | 0.417 | 8.75E-48 |
| ELOVL2-AS1 | EFCAB12 | 0.417 | 8.53E-48 |
| ELOVL2-AS1 | HMGN2P15 | 0.416 | 2.03E-47 |
| ELOVL2-AS1 | RIMS4 | 0.416 | 1.82E-47 |
| ELOVL2-AS1 | STMN1P1 | 0.416 | 2.18E-47 |
| ELOVL2-AS1 | EZH1 | 0.416 | 2.08E-47 |
| ELOVL2-AS1 | AL034374.1 | 0.416 | 2.48E-47 |
| ELOVL2-AS1 | MINDY4B | 0.415 | 4.43E-47 |
| ELOVL2-AS1 | IQCH | 0.415 | 4.12E-47 |
| ELOVL2-AS1 | AP1AR | 0.415 | 3.28E-47 |
| ELOVL2-AS1 | FAM13B | 0.415 | 3.39E-47 |
| ELOVL2-AS1 | NCAM2 | 0.415 | 4.18E-47 |
| ELOVL2-AS1 | GSTM3 | 0.415 | 2.65E-47 |
| ELOVL2-AS1 | TRIM66 | 0.415 | 3.57E-47 |
| ELOVL2-AS1 | CPEB2 | 0.414 | 4.67E-47 |
| ELOVL2-AS1 | KRT37 | 0.414 | 4.69E-47 |
| ELOVL2-AS1 | LZTFL1 | 0.413 | 8.72E-47 |
| ELOVL2-AS1 | FOXN1 | 0.413 | 1.01E-46 |
| ELOVL2-AS1 | MIR4429 | 0.413 | 8.25E-47 |
| ELOVL2-AS1 | CXorf40A | 0.413 | 8.47E-47 |
| ELOVL2-AS1 | RAD17 | 0.412 | 1.96E-46 |
| ELOVL2-AS1 | NAGS | 0.412 | 2.24E-46 |
| ELOVL2-AS1 | LINC00475 | 0.412 | 2.07E-46 |
| ELOVL2-AS1 | POTEI | 0.412 | 1.75E-46 |
| ELOVL2-AS1 | IGFBP4 | 0.412 | 1.98E-46 |
| ELOVL2-AS1 | AC092162.2 | 0.411 | 2.64E-46 |
| ELOVL2-AS1 | SEMA3B | 0.411 | 3.91E-46 |
| ELOVL2-AS1 | ATP6AP1L | 0.411 | 2.77E-46 |
| ELOVL2-AS1 | MDM2 | 0.411 | 3.00E-46 |
| ELOVL2-AS1 | ACOT4 | 0.411 | 3.57E-46 |
| ELOVL2-AS1 | AC008771.1 | 0.41 | 6.42E-46 |
| ELOVL2-AS1 | AF127577.4 | 0.41 | 6.66E-46 |
| ELOVL2-AS1 | SALL2 | 0.41 | 5.34E-46 |
| ELOVL2-AS1 | CIPC | 0.41 | 4.47E-46 |
| ELOVL2-AS1 | ZFP14 | 0.41 | 5.75E-46 |
| ELOVL2-AS1 | AC008124.1 | 0.41 | 4.52E-46 |
| ELOVL2-AS1 | AC100793.4 | 0.41 | 4.70E-46 |
| ELOVL2-AS1 | MAST4 | 0.41 | 5.73E-46 |
| ELOVL2-AS1 | AC120498.10 | 0.41 | 5.66E-46 |
| ELOVL2-AS1 | LINC01948 | 0.41 | 4.28E-46 |
| ELOVL2-AS1 | RBM11 | 0.409 | 7.26E-46 |
| ELOVL2-AS1 | CALCOCO1 | 0.409 | 8.05E-46 |
| ELOVL2-AS1 | RAB11FIP3 | 0.409 | 8.82E-46 |
| ELOVL2-AS1 | ADD1 | 0.409 | 7.74E-46 |
| ELOVL2-AS1 | AP003080.1 | 0.408 | 1.48E-45 |
| ELOVL2-AS1 | SAMD15 | 0.408 | 1.45E-45 |
| ELOVL2-AS1 | DACH1 | 0.407 | 2.99E-45 |
| ELOVL2-AS1 | PALM | 0.407 | 2.47E-45 |
| ELOVL2-AS1 | CCNG2 | 0.407 | 3.49E-45 |
| ELOVL2-AS1 | BMERB1 | 0.407 | 3.12E-45 |
| ELOVL2-AS1 | AL118522.1 | 0.407 | 2.15E-45 |
| ELOVL2-AS1 | TUBA3E | 0.407 | 2.20E-45 |
| ELOVL2-AS1 | MBOAT1 | 0.406 | 5.93E-45 |
| ELOVL2-AS1 | CFAP43 | 0.406 | 5.46E-45 |
| ELOVL2-AS1 | MEIS3P2 | 0.406 | 5.41E-45 |
| ELOVL2-AS1 | P4HTM | 0.406 | 4.03E-45 |
| ELOVL2-AS1 | DCTN4 | 0.406 | 4.77E-45 |
| ELOVL2-AS1 | UBE3B | 0.406 | 4.30E-45 |
| ELOVL2-AS1 | NTAN1P2 | 0.406 | 3.58E-45 |
| ELOVL2-AS1 | CCDC196 | 0.406 | 4.94E-45 |
| ELOVL2-AS1 | VN1R53P | 0.405 | 6.23E-45 |
| ELOVL2-AS1 | DPY19L2P4 | 0.405 | 7.29E-45 |
| ELOVL2-AS1 | BTF3 | 0.405 | 9.55E-45 |
| ELOVL2-AS1 | FOXA1 | 0.405 | 6.28E-45 |
| ELOVL2-AS1 | AC108134.4 | 0.405 | 6.17E-45 |
| ELOVL2-AS1 | BBS5 | 0.405 | 9.25E-45 |
| ELOVL2-AS1 | FAM86B3P | 0.405 | 8.71E-45 |
| ELOVL2-AS1 | CFAP70 | 0.404 | 1.33E-44 |
| ELOVL2-AS1 | AL645924.1 | 0.404 | 1.23E-44 |
| ELOVL2-AS1 | ZBTB7A | 0.404 | 1.35E-44 |
| ELOVL2-AS1 | HACD3 | 0.404 | 1.36E-44 |
| ELOVL2-AS1 | ZNF563 | 0.404 | 1.38E-44 |
| ELOVL2-AS1 | BTG2 | 0.404 | 1.74E-44 |
| ELOVL2-AS1 | ACER2 | 0.404 | 1.38E-44 |
| ELOVL2-AS1 | GEMIN7-AS1 | 0.403 | 2.87E-44 |
| ELOVL2-AS1 | BAIAP3 | 0.403 | 2.46E-44 |
| ELOVL2-AS1 | ERBB4 | 0.403 | 1.91E-44 |
| ELOVL2-AS1 | SPATA46 | 0.403 | 2.87E-44 |
| ELOVL2-AS1 | EXOC2 | 0.402 | 4.14E-44 |
| ELOVL2-AS1 | INSYN2A | 0.402 | 5.12E-44 |
| ELOVL2-AS1 | AC008770.3 | 0.402 | 4.48E-44 |
| ELOVL2-AS1 | MPP7 | 0.402 | 3.31E-44 |
| ELOVL2-AS1 | AC010326.5 | 0.402 | 4.50E-44 |
| ELOVL2-AS1 | ZNF20 | 0.402 | 3.53E-44 |
| ELOVL2-AS1 | ZNF680 | 0.402 | 3.32E-44 |
| ELOVL2-AS1 | TBX3 | 0.402 | 3.96E-44 |
| ELOVL2-AS1 | C9orf64 | 0.402 | 3.98E-44 |
| ELOVL2-AS1 | EMX1 | 0.402 | 3.50E-44 |
| ELOVL2-AS1 | TAPT1 | 0.402 | 3.71E-44 |
| ELOVL2-AS1 | ZSWIM5 | 0.402 | 5.07E-44 |
| ELOVL2-AS1 | ZNF587B | 0.402 | 3.66E-44 |
| ELOVL2-AS1 | GOLGA2P5 | 0.402 | 4.29E-44 |
| ELOVL2-AS1 | AP000821.1 | 0.402 | 4.47E-44 |
| ELOVL2-AS1 | AL691432.1 | 0.401 | 7.71E-44 |
| ELOVL2-AS1 | POC1B-AS1 | 0.401 | 6.40E-44 |
| ELOVL2-AS1 | RALGPS1 | 0.401 | 6.21E-44 |
| ELOVL2-AS1 | NUDT12 | 0.401 | 7.01E-44 |
| ELOVL2-AS1 | GLS2 | 0.401 | 6.99E-44 |
| ELOVL2-AS1 | METTL15 | 0.401 | 8.02E-44 |
| ELOVL2-AS1 | FAM184B | 0.401 | 7.54E-44 |
| ELOVL2-AS1 | AC114501.1 | 0.401 | 7.98E-44 |
| ELOVL2-AS1 | TMEM101 | 0.401 | 7.98E-44 |
